# Vitreal Concentrations of Vascular Endothelial Growth Factor in Patients with Rhegmatogenous Retinal Detachment

**DOI:** 10.3390/jcm12041259

**Published:** 2023-02-05

**Authors:** Hossein Hasanpour, Maria Cristina Kenney, Baruch D. Kuppermann, Mohammad Riazi Esfahani, Mozhgan Rezaei Kanavi, Mithalesh Kumar Singh, Masoud Soheilian

**Affiliations:** 1 Gavin Herbert Eye Institute, Department of Ophthalmology, University of California Irvine, Irvine, CA 92697, USA; 2 Mitochondrial Research, Gavin Herbert Eye Institute Ophthalmology, University of California Irvine, Irvine, CA 92697, USA; 3 Ocular Tissue Engineering Research Center, Research Institute for Ophthalmology and Vision Science, Shahid Beheshti University of Medical Sciences, Tehran 15167-45811, Iran; 4 Ophthalmic Research Center, Labbafinejad Medical Center, Shahid Beheshti University of Medical Sciences, Tehran 15167-45811, Iran

**Keywords:** VEGF, rhegmatogenous retinal detachment, proliferative vitreoretinopathy, ELISA

## Abstract

The purpose of this study is to evaluate the concentration of vascular endothelial growth factor (VEGF) in the vitreous humor of patients with primary rhegmatogenous retinal detachment (RRD). This is a prospective case control study. Eighteen patients with primary RRD without proliferative vitreoretinopathy C (PVR C) were enrolled as cases, and twenty-two non-diabetic retinopathy patients who were candidates for complete pars plana vitrectomy due to Macular Hole or Epiretinal Membrane were included as the control group. Undiluted vitreal samples were collected during the initiation of Pars Plana Vitrectomy (PPV) prior to any infusion into the posterior cavity. Vitreous samples were also collected from 21 fresh cadaveric globes. The vitreous concentration of VEGF was measured by enzyme-linked immunosorbent assay (ELISA) technique and compared between these two groups. The vitreal concentration of VEGF was 0.643 ± 0.088 ng/mL in the RRD group. Measured concentrations of VEGF in controls were 0.043 ± 0.104 ng/mL, and in cadaveric eyes they were 0.033 ± 0.058 ng/mL. The mean VEGF concentration in the RRD group was statistically higher than in the control group (*p* < 0.0001) and cadaveric eyes (*p* < 0.0001). Our study shows that vitreal VEGF concentrations significantly increase in patients with RRD.

## 1. Introduction

The potentially blinding condition known as rhegmatogenous retinal detachment (RRD) occurs when vitreous fluid leaks through a tear in the retina and into the space between the sensory retina and the retinal pigment epithelium (RPE). Proliferative vitreoretinopathy (PVR), which is the proliferation, expansion, and contraction of cellular membranes within the vitreous cavity and on retinal surfaces, is the leading cause of anatomic failure following initially successful retinal detachment surgery [1]. Retinal procedures, such as retinal cryopexy, laser retinopexy, pneumatic retinopexy, scleral buckle, and/or pars plana vitrectomy, can lead to PVR, as can untreated eyes with chronic retinal detachment. Although estimates of PVR prevalence vary widely between published series, it is generally believed that between 5 and 10% of RRD cases will ultimately develop PVR [2].

In order to better predict prognosis and direct surgical strategy, classification schemes for PVR have been developed [3]. Vitreous haze and/or pigment clumps in the vitreous cavity or on the inferior retina characterize a grade A diagnosis. Retinal tearing, retinal stiffness, vessel tortuosity, and/or rolled edges of retinal breaks indicate a grade B diagnosis. Retinal folds or subretinal bands spanning the entire retina are indicative of a grade C diagnosis [4].

Vascular endothelial growth factor (VEGF) plays an important role in retinal proliferative diseases. Eyes with retinal detachment and higher grades of PVR have also been linked to elevated VEGF levels [5,6,7,8].

VEGF levels have been found to be elevated in tissues associated with PVR [9]. Pennock et al. hypothesized that VEGF is a vulnerability of PVR due to the fact that it allows for PDGFR activation independent of PDGF. It is reasonable to consider anti-VEGF medications as a potential modality for preventing PVR given the role of VEGF in the pathogenesis of PVR. One’s findings suggests that ranibizumab, a vascular endothelial growth factor A (VEGF-A) inhibitor, was able to prevent PVR in an animal model [10]. Preliminary results from our study of intravitreal injection of Bevacizumab in primary vitrectomy to reduce the rate of retinal redetachment showed no improvement or worsening of anatomic or visual outcomes [11]. The results of our previous research prompted us to investigate whether or not VEGF levels are elevated in human RRDs and, if so, by how much compared to non-RRD conditions. We aimed to examine the vitreal VEGF concentration in patients with primary RRD by comparing the levels in cases without PVR C. Our secondary objective is to examine whether or not VEGF levels are correlated with break characteristics, such as type, location, and extension, in patients with primary RRD.

## 2. Materials and Methods

### 2.1. Ethical

The study complied with the guidelines of the Helsinki Declaration, and the methods were approved by the Institutional Review Board of the Shahid Beheshti University of Medical Sciences’ Ophthalmic Research Center in Tehran, Iran.

### 2.2. Study Design and Inclusions of Patients

Primary RRD cases and controls enrolled prospectively in this study. Inclusion criteria were having primary Rhegmatogenous Retinal Detachment (RRD) without PVR C, which are candidates for pars plana vitrectomy (PPV). All patients were over 18 years of age. Subjects with a history of any kind of detachment surgery, history of trauma or uveitis, diabetic retinopathy, diabetes mellitus, bleeding diathesis, hepatic or renal failure, age-related macular degeneration, and giant retinal tear were excluded from the study. All patients in both groups underwent a standard 3-port 23-gauge PPV.

### 2.3. Sample Collection

Undiluted vitreous sampling by vitrectomy probe was performed before opening the inflow port; sample volumes ranged from 0.5 to 0.7 mL and were immediately frozen and stored at −80 °C. We also analyzed the vitreous VEGF level in 22 non-diabetic retinopathy patients who were candidates for 23-gauge PPV due to Macular Hole (MH) or Epiretinal Membrane (ERM). We also analyzed the vitreous VEGF level in 21 fresh cadaveric globes.

### 2.4. Enzyme-Linked Immunosorbent Assay (ELISA)

The vitreous samples were sent for analysis of VEGF level with ELISA microplate reader (DYNEX technologies, MRX microplate reader, Chantilly, VA, USA). Each assay was performed according to the manufacturer’s instructions. The VEGF concentrations are presented as the means and standard deviations (mean ± SD; ng/mL).

### 2.5. Statistical Analyses

Statistical analyses were performed using the GraphPad prism (Version 8.0 for Windows, GraphPad Software, San Diego, California USA). The following symbols were used: * indicates *p ≤* 0.05, ** indicates *p <* 0.01, *** indicates *p <* 0.001, and **** indicates *p <* 0.0001; ns represents non-significant. One-way ANOVA using Sidak’s multiple comparisons test for comparison of mean VEGF in control, cadaveric, and RRD groups was used. The Mann–Whitney U test was applied to compare the VEGF level with the clinical parameters present or absent in the RRD group. Spearman’s Rho is a non-parametric test used to measure the strength of association between two variables, where the value r = 1 means a perfect positive correlation and the value r = −1 means a perfect negative correlation. Two-tailed probabilities of less than 0.05 were considered to indicate statistical significance.

## 3. Results

### 3.1. Demographic Details

In total, 18 patients with RRD, 22 control samples, and 21 fresh cadaveric globes were enrolled in this study. Table 1 fully describes the demographic features including sex, laterality, underlying disease, and lens status found in the control, cadaveric, and RRD groups. In the RRD group (Table 1), characteristics include extension, location, break number, and macula status (On, Off).

Cases and controls were sex-matched (13 males in cases and 10 males in controls, *p* = 0.229). Additionally, regarding the lens status, these two groups were matched (8 phakic and 10 pseudophakic in cases and 10 phakic and 12 pseudophakic in controls, *p* = 0.553). RRD cases and control were not age-matched, as the mean age in RRD cases wase significantly lower than controls (54.2 years vs. 66.1 years, *p* = 0.008). Patients’ ages ranged from 35 to 70 years old in the RRD group, while they ranged from 60 to 74 years old in the control or cadaveric group.

### 3.2. Expression of VEGF in the Control, Cadaveric, and Rhegmatogenous Retinal Detachment (RRD) Group

**Figure 1:**
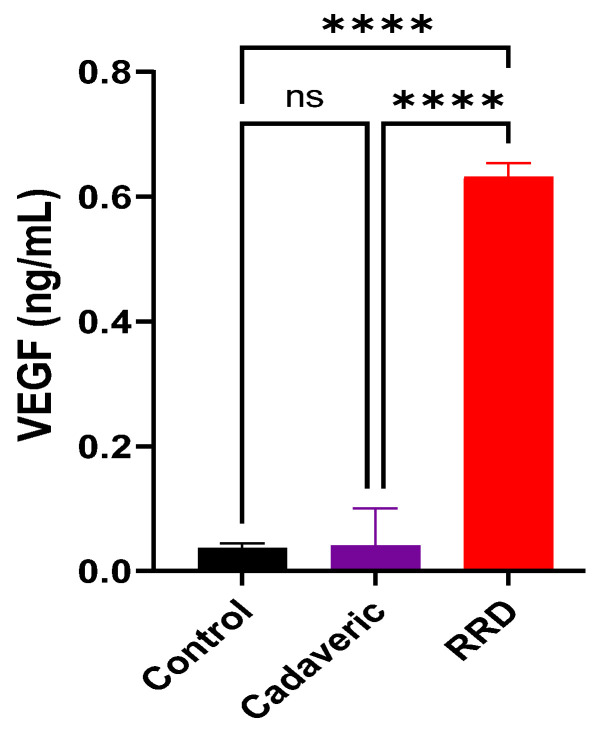
Mean expression level of VEGF (ng/mL) in the vitreous sample collected from the control, cadaveric, and RRD groups. Data shown as mean ± SD were analyzed by one-way ANOVA using Sidak’s multiple comparisons test. * indicates *p ≤* 0.05, ** indicates *p <* 0.01, *** indicates *p <* 0.001, and **** indicates *p <* 0.0001; ns represents non-significant.

The VEGF level in RRD group was statistically higher than control group (0.643 ± 0.088 vs. 0.043 ± 0.104, *p* < 0.001). The mean VEGF level in cadaveric group was similar to the control group (0.033 ± 0.058 vs. 0.043 ± 0.104, *p* ˃ 0.05) (Figure 1).

### 3.3. Neither the Type of Break nor the RRD Extension Affects the Levels of VEGF Expression

A Mann–Whitney U test was used to determine if VEGF concentrations were associated with the break extension, break number, break type, and age. Our data reveal that VEGF levels do not depend on the type or location of the breaks, older age, and sex. This was explained by the fact that no variables, including break type (Hole or Horseshoe tear), break size (or number), or break extension, were statistically significant (Table 2).

Spearman correlation was calculated to study the correlation between VEGF levels and RRD extension and break numbers (Table 3). We did not find any statistically significant relation between the VEGF level and RRD extension (r = −0.365, *p* = 0.147).

## 4. Discussion

Here, we show that patients with primary RRD without PVR C have a significantly higher vitreous level of VEGF compared to both controls and cadavers. PVR and the associated retinal traction are the primary causes of redetachment after the successful repair of retinal detachment. We also found that VEGF is detectable in the vitreous of patients with RRD, and that its level is higher than that of the normal group.

Although most cases of PVR are treatable through surgical intervention, the process of PVR and any subsequent detachments can cause significant damage to the retina, leading to poor visual outcomes even when successful. Although VEGF has been linked to PVR in a plethora of studies, its precise role in the pathogenesis of the condition remains unclear. The vitreous level of VEGF is significantly higher in PVR eyes compared to eyes with simple retinal detachment or macular hole, as discovered by Ogata et al. [12]_._ A two- to threefold increase in VEGF level was also reported by Ricker et al. in the PVR group, which is consistent with our findings [7]. Increased levels of VEGF may result from RRD-induced inflammation and oxidative stress, the breakdown of the blood–retinal barrier, and retinal hypoxia in cases of primary uncomplicated RRD [13].

Repeated studies in animal models and histological examinations of human retina specimens have established that the retinal pigment epithelium is the primary cellular component of PVR [14]. Retinal pigment epithelium cells are present in almost 100% of PVR membranes, and they may have different morphologic characteristics (macrophage-like, fibroblast-like) [13]. Retinal pigment epithelium (RPE) cells release VEGF in response to tissue damage [15,16]. We do not know whether VEGF derived from RPE immigrated through the break or RPE located at its residential area. To the best of our knowledge, there is no study reporting the VEGF level and break characteristics in eyes with RRD and PVR. Yet, we did not find any significant correlation between VEGF concentration and break length, break type, break location, or break number in the primary RRD patients. Similar to the findings of the Lahteenvuo group [17], we found no significant correlation between VEGF levels and older age in RRD patients. The limitation of our study is the small sample size and non-measurement of growth factors other than VEGF.

## 5. Conclusions

**Figure 2:**
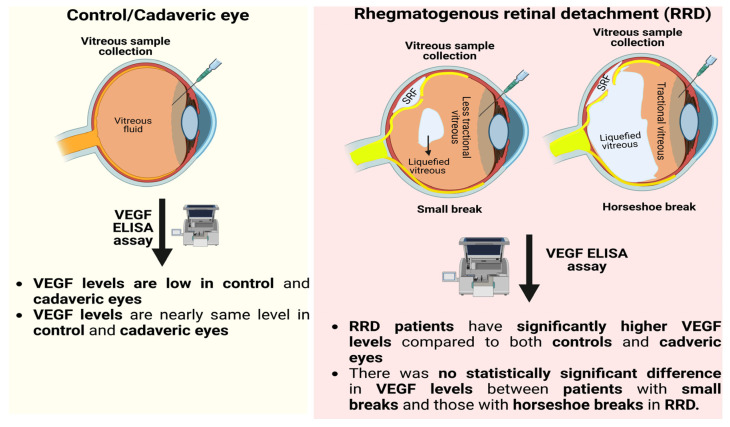
Schematic showing increased VEGF levels in the vitreous of patients with primary RRD compared to controls and cadaver eyes. In patients with primary RRD, the size of the break does not appear to have any effect on the patient’s VEGF levels. SRF represents sub-retinal fluid.

Our study showed that vitreous level of VEGF is significantly higher in patients with primary RRD than normal (Figure 2). It does not seem to be influenced by the break size or their numbers and may cast new information for future management of RRD and/or PVR.

## Figures and Tables

**Table 1:** Demographic features including sex, laterality, underlying disease, and retinal detachment characteristics found in the case, control, and cadaveric groups. Rhegmatogenous retinal detachment group including extension, location, break number, and macula status is shown. Number indicates number of cases in each group. Rhegmatogenous retinal detachment (RRD); Inferotemporal (IT); Inferonasal (IN); Supratemporal (ST); Supranasal (SN); Horseshoe tear (HST). Bold letter signifies statistically significant value.

Clinical Parameters		N (cases)	RRD	Control	Cadaveric	*p*-Value
Age (mean years)		61	54.2 years	66.1 years	61.8 years	0.008
Sex	Male	34 (56%)	13 (72%)	10 (46%)	11 (52%)	0.229
	Female	27 (44%)	5 (28%)	12 (54%)	10 (48%)	
Eye	OD	23 (58%)	9 (50%)	14 (64%)	0	0.398
	OS	17 (42%)	9 (50%)	8 (36%)	0	
Lens status	Phakic	29 (48%)	8 (44%)	21 (96%)	12 (57%)	0.553
	Pseudophakic	32 (52%)	10 (56%)	1 (4%)	9 (43%)	
Clinical details of rhegmatogenous retinal detachment (RRD)						
Clinical parameters		N (cases)	RRD			
Macular	On	1 (5.6%)	1 (5.6%)			
	Off	17 (94.4%)	17 (94.4%)			
RD extension (quadrant)	<1	2 (11.1%)	2 (11.1%)			
	1–2	5 (27.8%)	5 (27.8%)			
	2–3	2 (11.1%)	2 (11.1%)			
	>3	9 (50.0%)	9 (50.0%)			
RD. ST	No	3 (16.7%)	3 (16.7%)			
	Yes	15 (83.3%)	15 (83.3%)			
RD. SN	No	7 (38.9%)	7 (38.9%)			
	Yes	11 (61.1%)	11 (61.1%)			
RD.IT	No	4 (22.2%)	4 (22.2%)			
	Yes	14 (77.8%)	14 (77.8%)			
RD.IN	No	4 (22.2%)	4 (22.2%)			
	Yes	14 (77.8%)	14 (77.8%)			
RD	No	0 (0.0%)	0 (0.0%)			
	Yes	18 (100.0%)	18 (100.0%)			
Break C	1	7 (41.2%)	7 (41.2%)			
	>1	10 (58.8%)	10 (58.8%)			
Break extent (Disk diameter)	1	9 (56.3%)	9 (56.3%)			
	2	6 (37.5%)	6 (37.5%)			
	3	1 (6.3%)	1 (6.3%)			
Break.ST	No	10 (58.8%)	10 (58.8%)			
	Yes	7 (41.2%)	7 (41.2%)			
Break.SN	No	14 (82.4%)	14 (82.4%)			
	Yes	3 (17.6%)	3 (17.6%)			
Break.IT	No	8 (47.1%)	8 (47.1%)			
	Yes	9 (52.9%)	9 (52.9%)			
Break.IN	No	13 (76.5%)	13 (76.5%)			
	Yes	4 (23.5%)	4 (23.5%)			
Break. HST	No	9 (52.9%)	9 (52.9%)			
	Yes	8 (47.1%)	8 (47.1%)			
Break. Hole	No	5 (29.4%)	5 (29.4%)			
	Yes	12 (70.6%)	12 (70.6%)			
Break. Dialysis	No	15 (100.0%)	15 (100.0%)			
	Yes	0 (0.0%)	0 (0.0%)			

**Table 2:** VEGF levels relation to break location, extension, and number in the RRD vitrectomy samples. Data shown as mean ± SD were analyzed by Mann–Whitney U test.

Clinicopathological Parameters		VEGF Level (Mean ± SD)	*p*-Value	Post Hoc Power (φ)
Age	<45 yrs	0.605 ± 0.033	0.51	26.5%
	≥45 yrs	0.648 ± 0.098		
Sex	Male	0.244 ± 0.294	0.52	5.1%
	Female	0.194 ± 0.296		
Eye	OD	0.304 ± 0.308	0.72	4.3%
	OS	0.341 ± 0.339		
Break extent (disk diameter)	1	0.67 ± 0.101	0.59	25.8%
	2	0.632 ± 0.074		
	3	0.59 ± 0		
Break.ST	No	0.618 ± 0.072	0.17	26.3%
	Yes	0.68 ± 0.108		
Break.SN	No	0.651 ± 0.098	0.49	20.1%
	Yes	0.61 ± 0.044		
Break.IT	No	0.673 ± 0.106	0.23	23.6%
	Yes	0.618 ± 0.071		
Break.IN	No	0.664 ± 0.093	0.09	72.6%
	Yes	0.578 ± 0.043		
Break.HST	No	0.632 ± 0.067	0.60	3.2%
	Yes	0.656 ± 0.116		
Break.Hole	No	0.68 ± 0.137	0.3	12.5%
	Yes	0.628 ± 0.065		
Break number	1	0.664 ± 0.123	0.74	5.1%
	2	0.593 ± 0.058		
	3	0.666 ± 0.062		
	4	0.59 ± 0		
	5	0.59 ± 0		

**Table 3:** Spearman Correlation Coefficient Analysis.

Spearman’s Rho		VEGF
RD Extension (quadrant)	Correlation Coefficient	−0.356
	Sig. (2-tailed)	0.147
	N	18
Break Number	Correlation Coefficient	−0.028
	Sig. (2-tailed)	0.914
	N	17
Break Extension (disc diameter)	Correlation Coefficient	−0.262
	Sig. (2-tailed)	0.327
	N	16

## Data Availability

Not applicable.

## Abbreviations

Rhegmatogenous retinal detachment (RRD); Proliferative vitreoretinopathy (PVR); Enzyme-linked immunosorbent assay (ELISA); Pars plana vitrectomy (PPV); Inferotemporal (IT); Inferonasal (IN); Supratemporal (ST); Supranasal (SN); Horseshoe tear (HST).
